# "Peer2Peer" – A university program for knowledge transfer and consultation in dealing with psychosocial crises in med-school and medical career

**DOI:** 10.3205/zma001051

**Published:** 2016-08-15

**Authors:** Christian Vajda

**Affiliations:** 1 Medical University of Graz, Department of Medical Psychology and Psychotherapy, Graz, Austria

**Keywords:** psychosocial support systems, medical students

## Abstract

**Objective: **Medical students are exposed to various psychosocial problems and challenges. Specific consultations services and programs can support them.

“Peer2Peer” is such a consultation program and was implemented at the Medical University of Graz. It focusses on crisis intervention, psychosocial stress management, junior mentoring as well as student education in this field. Besides, it also offers student tutors of the program practical skills trainings. The program was restructured in winter term 2014/15.

**Methods: **On the one hand, “Peer2Peer” gives insights into topics such as the current state of research concerning the students’ psychological strain and psychosocial crises in acutely stressful situations and preventive approaches for coping with these kinds of situations on the other hand. These aspects are taught by means of elective courses, lectures and workshops. Furthermore, “Peer2Peer” provides consultation services by student tutors who give face-to-face advice if required. These tutors receive ongoing training in organizational and professional issues.

**Results:** Since the summer term of 2015, 119 students have been trained (via lectures and elective courses), while 61 contacts (short consultation) and 33 contacts (full consultation) have been supervisied. In total, two psychotherapeutic and one psychosocial follow ups were recommended. There are seven students who participate as tutors in the program.

**Conclusions:** The “Peer2Peer” program is intended to enable a low-threshold access for medical students facing psychosocial crises situations and to help them in dealing with stress and learning problems. An increase in support contacts from the summer term of 2015 to the winter term of 2015/16 can be considered a success. A first evaluation of the different components of the program started in the winter semester of 2015/16. The student tutors have not only acquired practical skills in dealing with students in crises situations but also various organizational skills.

## Zusammenfassung

**Zielsetzung: **Medizinstudierende sind vielfältigen psychosozialen Belastungen und Herausforderungen ausgesetzt. Spezifische Beratungs- und Betreuungsprogramme können eine Unterstützungsfunktion innehaben. An der Medizinischen Universität Graz nimmt das „Peer2Peer“-Programm diese Aufgabe im Zuge eines Angebotes zur Krisenintervention, psychosozialen Stressbewältigung, Junior-Mentoring, Fort- und Weiterbildung für Studierende als auch praktischer Kompetenzsteigerung der betreuenden Tutor/-innen wahr. Mit Wintersemester 2014/15 erfolgte eine Neustrukturierung.

**Methodik: **Die Vermittlung von Themen wie der Evidenz zur Belastung von Studierenden, Umgang mit psychosozialen Krisen im akuten Fall sowie präventive Ansätze erfolgt über ein Wahlfach, einen Beratungsdienst, besetzt durch studentische Tutor/-innen, sowie spezifische Fort- und Weiterbildungen. Die Tutor/-innen werden laufend in organisatorischen und fachlichen Belangen geschult.

**Ergebnisse:** Seit dem Sommersemester 2015 wurden 119 Studierende in themenspezifischen Veranstaltungen (Vorträge und Wahlfach) weitergebildet und 61 kurze sowie 33 ausführliche Beratungskontakte durchgeführt. Zwei psychotherapeutische und eine psychosoziale Weitervermittlungen wurden veranlasst. Sieben Studierende nehmen als Tutor/-innen am Programm teil.

**Schlussfolgerungen:** Das Peer2Peer-Programm soll einen niederschwelligen Zugang für Medizinstudierende in psychosozialen Krisensituationen sowie bei Fragen zur Stressbewältigung und Lernproblemen ermöglichen. Eine Steigerung der Betreuungskontakte von Sommersemester 2015 auf Wintersemester 2015/16 kann als Erfolg gewertet werden. Eine erste Teilevaluierung der unterschiedlichen Programmbestandteile ist seit dem Wintersemester 2015/16 in Durchführung. Für die studentischen Tutor/-innen ist ein Wissenszuwachs bezüglich des praktischen Umganges mit Personen in Krisensituationen sowie in diversen organisatorischen Kompetenzen zu vermerken.

## Introduction

Medical students are facing many challenges during medical school. Among others, these include dealing with topics like life and death, illness and suffering, the perception one’s physical and emotional limitations, problems in private life and financial matters. Consequences could result in a higher burden of anxiety and depression [1], [2] and a lower quality of life in comparison to students of other disciplines and the general population [3], respectively. Substantive and structural conditions of the curricula can favor the occurrence of these burdens [4], [5] and even intensify them over time [6]. 

Experiences made at medical school generate an important impact for the students’ future work-life balance. Thus, positive and negative stress management is highly important [7], [8], [9], [10], [11]. The effects of inadequate regulation skills can result in health risks for patients, such as a higher rate of prescription errors of depressed and burnout physicians [12]. 

An international comparison indicates a large diversity of support services for students [13], [14].These include lectures (including behavior change strategies and evidence-based data on psychosocial strain) and tutorials (exercises for mindfulness and group discussions) in the core curriculum like the Health Enhancement Program (HEP) at Monash University in Australia [15] or consultation programs at the Eastern Virginia Medical School. In this school, medical students can discuss experiences of psychosocial stress and receive medical care from a medical consultant of the department of “Family and Community Medicine” at the beginning and at the end of the academic year [16]. Some universities even involve their students in the development and design of such programs [17].

By implementing the “Peer2Peer” program, the Medical University of Graz strives to increase the students’ awareness for matters of psychosocial stress during their studies and their future professional life. Furthermore, the program supports students in coping with challenges in their academic and private life and aims at increasing knowledge in dealing with patients who are experiencing extreme psychosocial situations.

## Description of the program

The “Peer2Peer” program focuses on topics such as crisis intervention, psychosocial stress management, knowledge transfer and junior mentoring free of charge for all students (n>4000). The program was restructured in the winter term of 2014/15. Consultation services and public relation activities started in the summer term of 2015.

The four aforementioned main topics are realized by three organizational components of the program:

1. An elective course open to all students
2. Support in crises and psychosocial stress situations as well as junior mentoring by student tutors (peers) through consultation services
3. Practical and theoretical training (lectures and workshops)

### Elective courses and training of the tutors

An elective course (2 terms) was implemented for training the tutors and transferring knowledge to the students in general. Students of all courses of study at the Medical University of Graz are entitled to participate (“Psychosocial crisis intervention and stress management I and II”, each 1 ECTS). The interdisciplinary staff (n=4, psychiatry, psychology, psychotherapy) is highly experiences in teaching, research and patient care. The five course units for each term (5x135 minutes) include the following topics:

#### Winter term

1. Introduction to psychosocial stress and resources for students, current state of research, general information about the “Peer2Peer” program
2. New in 2015/16: relaxation techniques (overview and practical exercises)
3. Basics in psychotherapeutic topics
4. Basics in psychiatric topics (i.e.: depression, anxiety)
5. Detailed information about “Peer2Peer”, case reports of “Students in psychosocial crises”, reflection on the term (feedback in group discussions/written work) and preparation for the following term

##### Summer term

1. Challenges in the supervision of students (case studies and current evidence)
2. Relaxation techniques/coping mechanisms/resources strengthening
3. Case studies from psychotherapeutic practice, role plays
4. Specific psychiatric topics (handling suicidality)
5. Summary of the acquired knowledge, Q&A on the “Peer2Peer” program, information about support networks, reflection

Pre-course requirement for the participation in this elective is the completion of the fourth academic term. The performance assessment is carried out by a. a compulsory attendance of 80%, b. active participation and c. a written reflection at the end of the course.

#### Structure of the consultation service

After successfully completing both parts of the elective, students are allowed to work as tutors (1 to 3 hours per week; October to January and March to June) as part of the consultation service of the university. The tutors offer at least two consultation time slots per week (Monday and Wednesday, 3pm to 4:30pm) and they can be contacted by the students via email, Facebook and telephone. Consultations can also be arranged in addition to the two fixed time slots. A room is constantly blocked for the tutors. Tutors mostly deal with the following topics: developing a schedule for revising study contents, psychosocial crises in the private life, introduction to relaxation techniques, and / or coping with exam stress. An advisory board for more complex issues (i.e., psychotherapeutic follow-up care) was set up at the Department of Medical Psychology and Psychotherapy. Students can use the consultation service free of charge and anonymously.

**Figure 1:**
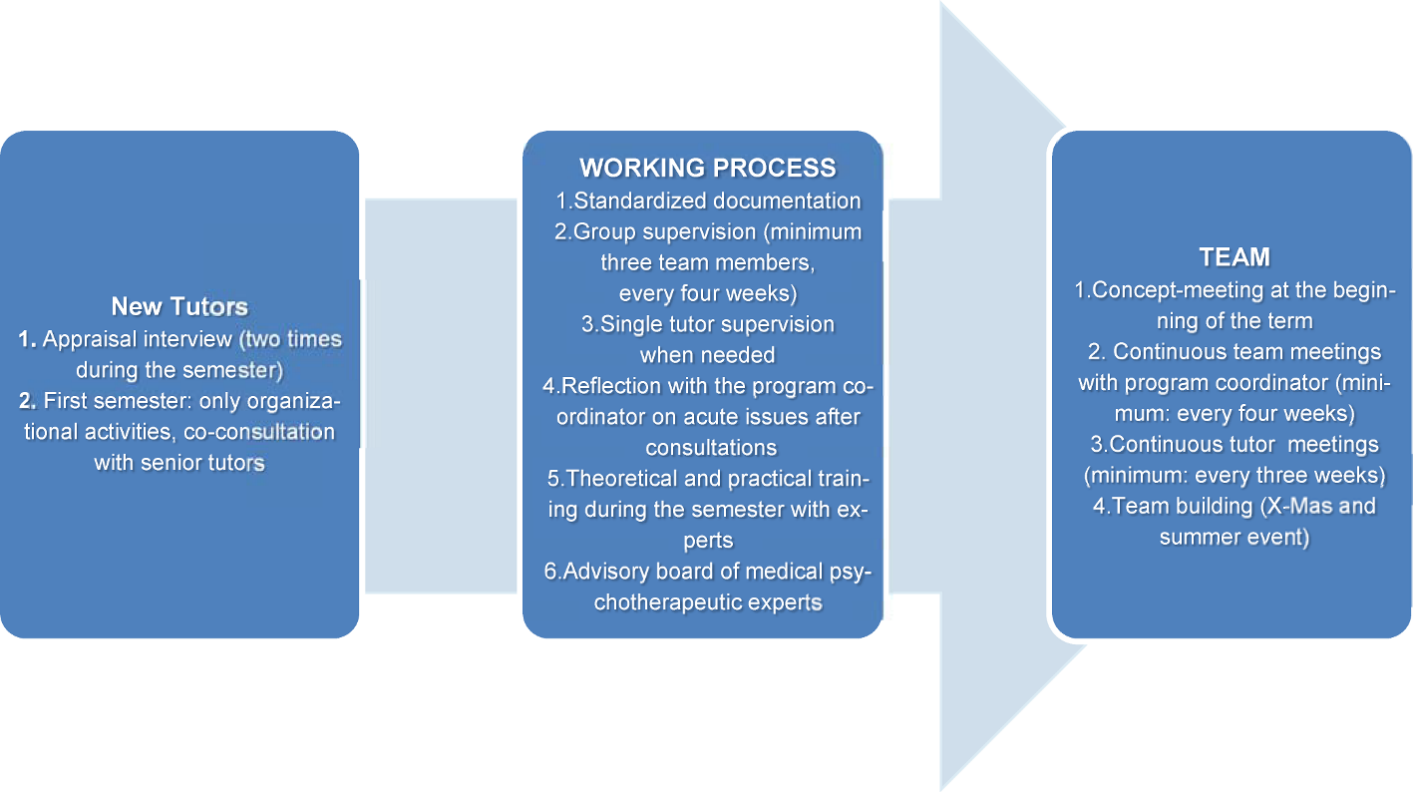
Overview of the three pillars of quality management of the “Peer2Peer” program at the Medical University of Graz

The quality management (including: tutor training, team meetings, appraisal interview with tutors, standardized documentation, supervisions) is shown in figure 1 (Fig. 1).

#### Training

In addition to consultation in psychosocial, study-related crises cases and junior mentoring, a focus is put on prevention and health promotion since winter term 2014/15. For this purpose, the tutors and the program coordinator organize lectures and workshops, information sessions and analyze structure-related problems (i.e., lack of recreation options) of the study program.

## Results

### Report of performance

In summer term 2015, five students worked in the program. In winter term 2015/16, seven students participated as tutors in the consultation service. Two new tutors who had previously completed the elective helped for purposes of quality management only in organizational activities (see figure 1 (Fig. 1)).

The consultation (see table 1 (Tab. 1)) and knowledge transfer (see table 2 (Tab. 2)) increased from the summer term (starting after restructuring in winter 2014/15) to the winter term of 2015/2016. Exam stress, distress because of board exam, fears of failure, family conflicts and difficulties in learning are the main problems addressed in the consultation service. Lectures and workshop, focused on the topics "relaxation" and "learning strategies", were open to all students of the university.

#### Elective

After restructuring the program in the winter term of 2014/15, the number of participants remained at a comparable level in winter term 2015/16 (see table 2 (Tab. 2)). Due the lack of written reflections (third performance criteria), the final result for the winter term of 2015/16 is not available yet. Thus, no final summary can be drawn at the time of submitting this article. However, 15 students meet the necessary requirements (compulsory attendance and active participation). The drop in the numbers of students from winter to summer term and within the two terms can have various reasons. An essential aspect could be the often simultaneously held obligatory core curriculum courses. That makes it impossible for several students to achieve the necessary attendance of at least 80%. The fact that the first part is obligatory for the second part of the elective usually leads to a smaller number of participants compared to the start of the winter term.

#### Practical skills and knowledge transfer for tutors

In addition to the acquisition of theoretical knowledge, insights into backgrounds and practical strategies for dealing with psychosocial crises (assessment, different approaches of psychotherapy and psychiatry, helping networks, correct referrals for future work, dealing with suicidal tendencies, accompaniment of students, professional interviewing) there is a focus in organizational skills (team coordination, rostering, meeting planning and execution etc.), event management (training for students) and public relations activities (representation, social media, etc.). Apart from that, tutors participate in at least two professional trainings (for example: relaxation techniques, behavioral approaches in the care of students, case discussions) during the academic term. As part of the quality management, they attend appraisal interviews as well as individual and group supervisions. 

## Discussion

Implementing the “Peer2Peer” program is an attempt to create a low-threshold consultation service focused on the needs of students. Due to the fact that there was no program at the Medical University of Graz before, a greater awareness for the topic “distress in academic studies and working life” was achieved on a local university level.

A complete restructuring of the program, including new content and organizational aspects (elective, consulting service, training), was carried out in the winter term of 2014/15. As a second step, the consultation service for students and reinforced public relations activities were launched in summer term 2015. The increase in the number of performances of consultation services from summer term 2015 to winter term 2015/16 can be considered as a first success of the efforts to raise the students’ awareness for the program.

In a third step, the evaluation of the program components was launched in the winter term of 2015/16. At the time of editing this article, the evaluation of the first part of the elective had not been completed yet. A publication is planned for the GMA conference 2016th. An evaluation of the tutors’ activities, an acceptance analysis of the program and a survey focusing on the needs of students is planned for 2016.

Based on past experiences, intense public relations activities (among others: university news feed, posters and flyers, students manuals) are necessary in order to reinforce the awareness of students for the program and to implement it as a permanent consultation service. Further topics (practical year, board exams, internship, etc.) and more consultation time slots are planned for the academic year of 2015/16.

## Contact details of the program

**Website:** http://www.medunigraz.at/peer2peer

**Facebook:** http://www.facebook.com/peer2peer

**E-Mail:** peer2peer@medunigraz.at

## Acknowledgements

Hereby the author wants to express his heartfelt thanks to the dedicated teachers and student tutors of the program and to the institutional services of the university. He would also like to thank the stuff and departments of the study organization for providing the necessary financial support (employment of the tutors). Without them the implementation of the program would not be possible.

## Competing interests

The author declare that he has no competing interests.

## Figures and Tables

**Table 1:**
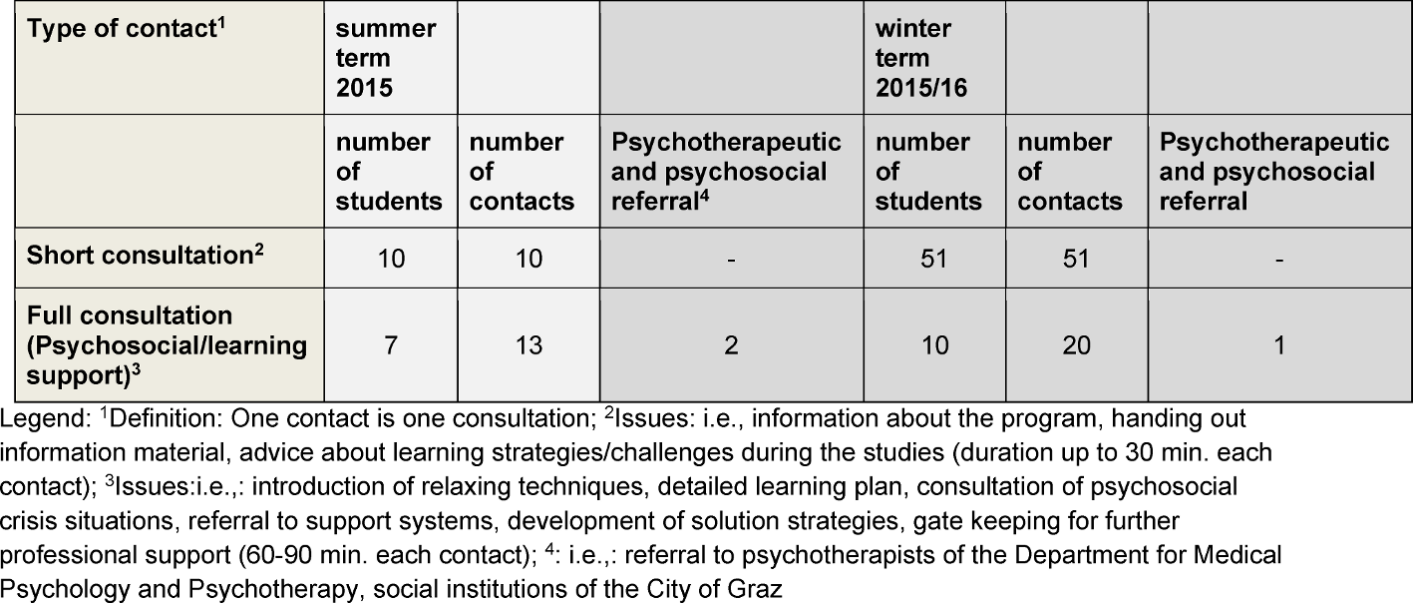
Table1: Overview of the number of consultation contacts of the „Peer2Peer“ program (February 2016)

**Table 2:**
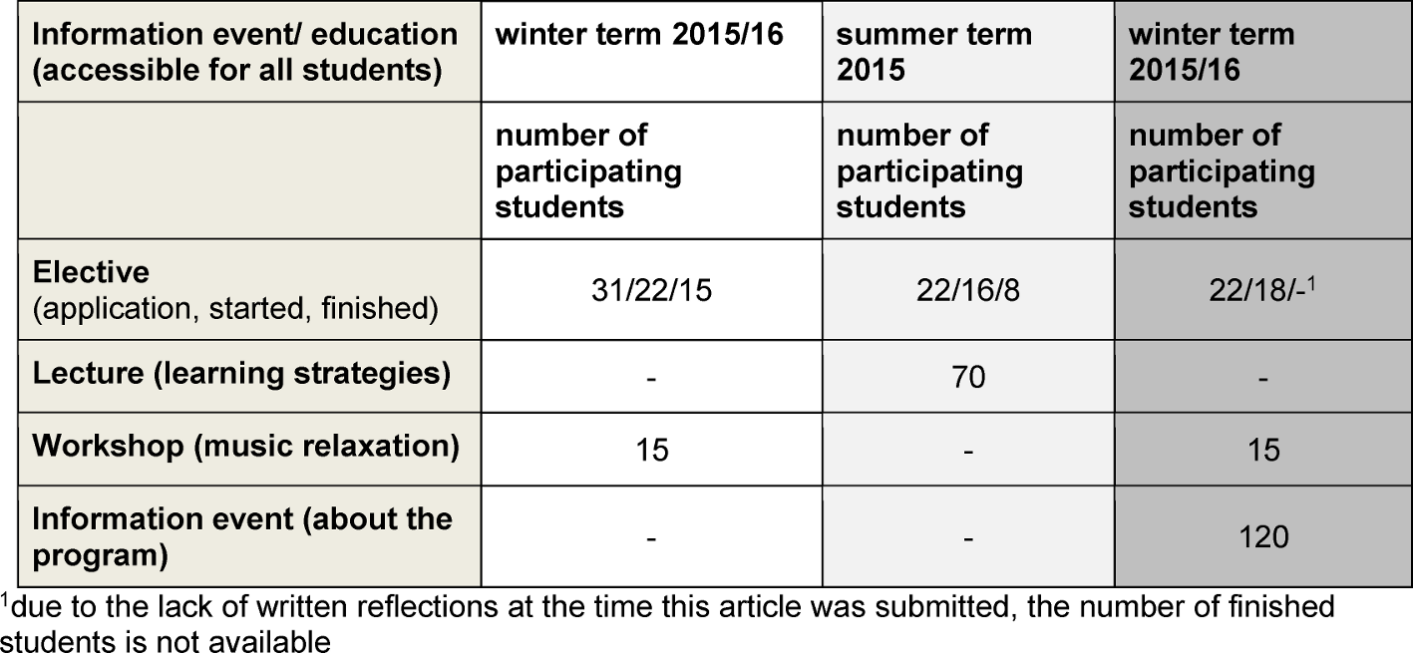
Overview of the number of participating students in educational activities of the program (without tutor trainings)
